# Safety considerations during return to work in the context of stable COVID-19 epidemic control: an analysis of health screening results of all returned staff from a hospital

**DOI:** 10.1017/S0950268820002150

**Published:** 2020-09-18

**Authors:** Ping Duan, Zhi-Qing Deng, Zhen-Yu Pan, Yan-Ping Wang

**Affiliations:** 1 Department of Orthopedics, Zhongnan Hospital of Wuhan University, Wuhan, 430071, Hubei, China; 2 Department of Medical Affairs, Zhongnan Hospital of Wuhan University, Wuhan, 430071, Hubei, China; 3 Department of Obstetrics and Gynecology, Hubei Provincial Hospital of TCM, Wuhan, 430061, Hubei, China

**Keywords:** Asymptomatic infection, COVID-19, return to work, SARS-CoV-2, screening

## Abstract

In March 2020, China had periodically controlled the coronavirus disease-19 (COVID-19) epidemic. We reported the results of health screening for COVID-19 among returned staff of a hospital and conducted a summary analysis to provide valuable experience for curbing the COVID-19 epidemic and rebound. In total, 4729 returned staff from Zhongnan Hospital of Wuhan University, Wuhan, China were examined for COVID-19, and the basic information, radiology and laboratory test results were obtained and systematically analysed. Among the 4729 employees, medical staff (62.93%) and rear-service personnel (30.73%) were the majority. The results of the first physical examination showed that 4557 (96.36%) were normal, 172 (3.64%) had abnormal radiological or laboratory test results. After reexamination and evaluation, four were at high risk (asymptomatic infections) and were scheduled to transfer to a designated hospital, and three were at low risk (infectivity could not be determined) and were scheduled for home isolation observation. Close contacts were tracked and managed by the Center for Disease Control and Prevention (CDC) in China. Asymptomatic infections are a major risk factor for returning to work. Extensive health screening combined with multiple detection methods helps to identify asymptomatic infections early, which is an important guarantee in the process of returning to work.

## Introduction

In December 2019, the severe acute respiratory syndrome coronavirus 2 (SARS-CoV-2) caused the coronavirus disease 2019 (COVID-19) outbreak in Wuhan, China. After a series of effective prevention, control and treatment measures were implemented, the epidemic of COVID-19 was curbed in a short time. Therefore, how to safely return to work has become the focus of attention. The outcome of SARS-CoV-2 infection depends largely on the interaction of the virus with the host, and theoretically, asymptomatic carriers may occur when the host's antiviral defense is too strong or uncoupled. Asymptomatic shedding may occur when the immune response effectively limits but does not completely prevent SARS-CoV-2 replication [1]. Asymptomatic SARS-CoV-2 carriers were first reported in a family clustering study [2], followed by an increasing number of reports of asymptomatic infections [3–5]. To avoid a re-epidemic of COVID-19, the health census of returned staff is important. In addition, returned staff are asymptomatic, and census is the most effective method to screen asymptomatic infections. Although some studies suggest that asymptomatic SARS-CoV-2 carriers may have weak infectivity [6], asymptomatic infections remain an important potential risk factor for re-epidemic. The combination of SARS-CoV-2 nucleic acid test with specific serological tests may help to detect asymptomatic subjects with SARS-CoV-2 infection [7]. We believe that effective detection of asymptomatic infections, standardised management and treatment and development of close contact tracking programmes can effectively control the COVID-19 epidemic.

## Materials and methods

### Study design

This is a descriptive study. From 16 March 2020 to 11 May 2020, a health screening for COVID-19 was conducted among all returned staff (roughly divided into three categories: medical staff, administrative staff and rear-service personnel) in the Zhongnan Hospital of Wuhan University, Wuhan, China.

According to the regulations of epidemic prevention and control, physical examination personnel must wear masks, queue up for sampling and related examinations according to the appointed time, the distance between personnel is more than 1 m, and doctors wear strict protective equipment, such as protective clothing, goggles, gloves, etc. The study was approved by the Medical Ethics Committee of Zhongnan Hospital of Wuhan University (no. 2020015), with a waiver of informed consent.

### Data collection

The study data were obtained from the database of the Physical Examination Report Center. Demographic information (age, sex and occupational category), chest computed tomography (CT) findings, antibody and SARS-CoV-2 RNA reverse transcription-polymerase chain reaction (RT-PCR) test results of all included subjects were obtained. Laboratory examination and chest CT scan results were analysed by three independent researchers.

### Laboratory examination

According to the protocol previously announced by the WHO, SARS-CoV-2 RNA was detected by real-time RT-PCR, and throat swabs were sampled from the subjects. The samples were sent to the clinical laboratory of the Zhongnan Hospital of Wuhan University for SARS-CoV-2 RNA detection. The antibodies against SARS-CoV-2 were detected by using an enzyme-linked immunosorbent assay based on the recombinant nucleocapsid protein of SARS-CoV-2. According to the manufacturers, the sensitivity and specificity are ~90% and >99% for immunoglobulin M (IgM), and ~98% and ~98% for IgG, respectively. Similarly, we collected blood samples and sent them to the Laboratory Department of Zhongnan Hospital of Wuhan University in time for antibody detection.

### CT imaging

CT scans are used to determine whether employees have viral pneumonia changes in their lungs. The positive criteria for CT screening were: (1) single, multiple or diffuse ground glass opacity (GGO), in which thickened blood vessels and thickened bronchial shadows were seen; (2) single or multiple solid shadows and (3) the original GGO or consolidation range increased and the number increased 3–5 days after the imaging suspected cases, or accompanied by pleural effusion on one side or both sides. Chest CT scanning was performed in the Imaging Department of Zhongnan Hospital of Wuhan University.

### Definition criteria and classification of abnormal results

Those with viral pneumonia change on chest CT scan or positive antibody (IgG or IgM) or positive nucleic acid test in the physical examination results were defined as abnormal population, and the people with abnormal results on the first physical examination should be reexamined. We set up a risk assessment expert group to give evaluation opinions based on the test results, including agreeing to return to work, postponing return to work, or transferring to a designated hospital for isolation treatment.

### Statistical analysis

All research objects employed fundamental descriptive analysis. Continuous variables were expressed as the medians and interquartile ranges (IQR). Categorical variables in each category were summarised as counts and percentages. Analysis of variance was used for comparison. *P* < 0.05 was considered statistically significant. Statistical analysis was performed using IBM SPSS software (version 23.0).

## Results

### Demographic characteristics

A total of 4729 subjects were included in the study. None of them had symptoms of respiratory infection (including cough, shortness of breath, nasal obstruction, etc.), and their body temperature were within the normal range (36.3–37.2°C). The male-to-female ratio in the total population is about 1:2, with a centralised age distribution between 18.0 and 60.0 years, with a median age of 33.0 (IQR: 28.0–47.0) years. Medical staff (62.93%) accounted for the largest proportion, followed by rear-service personnel (30.73%) and administrative staff (6.34%). The results of the first physical examination were normal in 4557 cases (96.36%) and abnormal in 172 cases (3.64%). In the population with abnormal physical examination results, the male-to-female ratio was about 2:3, with the highest proportion of persons aged 45.0 to 60.0 years old (34.88%), and the median age was 40.0 (IQR: 30.0–56.0) years. Detailed statistical characteristics of the study population are summarised in Table 1.

**Table 1.** Statistical characteristics of the subjects

	Total population		Normal outcome population		Abnormal outcome population	
	*N*	%	*N*	%	*N*	%
Gender						
Male	1578	33.37	1509	33.11	69	40.12
Female	3151	66.63	3048	66.89	103	59.88
Age (years)						
18–30	1876	39.67	1832	40.20	44	25.58
31–44	1485	31.4	1437	31.54	48	27.91
45–60	1161	24.55	1101	24.16	60	34.88
⩾61	207	4.38	187	4.10	20	11.63
Occupational category						
Medical staff	2976	62.93	2883	63.27	93	54.07
Administrative staff	300	6.34	289	6.34	11	6.4
Rear-service personnel	1453	30.73	1385	30.39	68	39.53
Total	4729	100	4557	100	172	100

### Laboratory and chest CT findings

Some subjects have recently undergone CT examination. The chest CT findings of the included subjects were divided into normal, viral pneumonia changes and non-viral pneumonia lesions. Due to the radiation hazards of CT scan, there were 2535 cases (53.61%) without chest CT examination. Among patients undergoing chest CT examination, 25 cases (0.53%) had viral pneumonia changes (Table 2).

**Table 2.** Distribution characteristics of laboratory examination results

	Total population		Abnormal outcome population	
	*N*	%	*N*	%
Chest CT finding				
Normal	2138	45.21	52	30.23
Viral pneumonia changes	25	0.53	25	14.53
Non-viral pneumonia lesions	31	0.66	31	18.02
Not done	2535	53.61	64	37.21
SARS-CoV-2 nucleic acid test				
Negative	4696	99.30	170	98.84
Positive	2	0.04	2	1.16
Not done	31	0.66	0	0
Detection of SARS-CoV-2 antibody				
IgG single positive	82	1.73	82	47.67
IgM single positive	23	0.49	23	13.37
Double positive	15	0.32	15	8.72
Double Negative	4609	97.46	52	30.23
Total	4729	100	172	100

According to the results of antibody detection, the subjects were divided into four groups: IgG single positive, IgM single positive, double positive and double negative. Among the people with abnormal physical examination results, 82 cases (47.67%) were IgG single positive, accounting for the highest proportion (Table 2). The average values of antibodies in different ages, sexes and occupations are shown in Table 3. There is no significant difference in the production of IgM and IgG antibodies among different ages, sexes and occupations.

**Table 3.** Distribution characteristics of SARS-CoV-2 antibody values among people with abnormal first physical examination results

	IgM antibody		IgG antibody	
	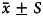	*P*	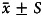	*P*
Age (years)				
18–30	16.33 ± 5.48	0.543	36.84 ± 22.52	0.202
31–44	18.62 ± 14.18		45.25 ± 30.31	
45–60	23.14 ± 16.19		50.47 ± 34.29	
⩾61	13.03 ± 1.78		58.11 ± 39.67	
Gender				
Male	23.31 ± 19.74	0.347	42.38 ± 28.86	0.369
Female	18.6 ± 10.7		48.3 ± 32.61	
Occupation				
Medical staff	19.24 ± 11.1	0.232	40.23 ± 28.35	0.221
Administrative staff	20.37 ± 10.72		54.86 ± 39.33	
Rear-service personnel	20.42 ± 16.78		50.59 ± 32.29	

There were 4698 cases (99.34%) of SARS-CoV-2 nucleic acid testing in the total population, of which two cases were positive in the initial SARS-CoV-2 nucleic acid testing and were asymptomatic infections. A total of 31 cases (0.66%) did not perform SARS-CoV-2 nucleic acid testing (Table 2), but they had recently accepted SARS-CoV-2 nucleic acid tests in other hospitals, and the results were all negative.

### Outcome events

**Fig. 1.**
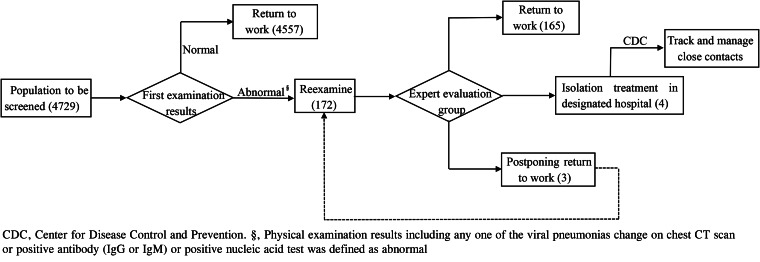
Physical examination and handling procedures.

In total, 4729 returned staff were screened by physical examination for the first time, 4557 (96.36%) normal people were allowed to return to work, and 172 cases (3.64%) with abnormal initial physical examination results were arranged for reexamination. After the discussion of the reexamination results by the expert evaluation group, 165 cases (95.93%) were allowed to return to work, four cases (2.33%) were transferred to the isolation treatment of Leishenshan hospital, three cases (1.74%) were recommended to stay at home and wait for the next reexamination (Fig. 1).

Among the four people transferred to Leishenshan Hospital for isolation treatment, two patients had positive initial SARS-CoV-2 nucleic acid test; one patient had negative initial SARS-CoV-2 nucleic acid test but showed positive after reexamination; the other one had negative multiple SARS-CoV-2 nucleic acid test, but had high SARS-CoV-2 antibody level, with IgG and IgM antibody levels of 100.35 and 37.96 AU/ml, respectively (normal IgM and IgG <10 AU/ml), and chest CT showed multiple ground-glass density patches in both lungs (Table 4).

**Table 4.** Summary of basic information, radiology and laboratory examination results of the seven cases who did not return to work

	People 1	People 2	People 3	People 4	People 5	People 6	People 7
Basic information							
Age (years)	31	68	63	57	45	58	61
Gender	Male	Male	Male	Female	Female	Female	Female
Occupation	Medical staff	Rear-service personnel	Rear-service personnel	Rear-service personnel	Rear-service personnel	Rear-service personnel	Rear-service personnel
Real-time RT-PCR							
The first time	+	+	−	−	−	−	−
The second time	NA	NA	+	NA	−	−	−
The third time	NA	NA	NA	NA	−	NA	NA
SARS-CoV-2 antibody							
IgM and its levels (AU/ml)	−	−	−	+; 37.96	+; 60.55	+; 19.62	−
IgG and its levels (AU/ml)	+; 16.27	+; 28.71	+; 89.22	+; 100.35	−	+; 100.01	+; 100.71
Radiological examination							
Chest CT findings	Nodules in the apex of the right lung	NA	Chronic inflammation of left pulmonary lingual lobe	Multiple ground glass density patches in both lungs	Secondary tuberculosis in posterior apical segment of upper lobe of left lung	NA	NA
Others							
Infectious risk	High-risk	High-risk	High-risk	High-risk	Low-risk	Low-risk	Low-risk
Outcome	Isolation treatment in designated hospital	Isolation treatment in designated hospital	Isolation treatment in designated hospital	Isolation treatment in designated hospital	Postponing return to work	Postponing return to work	Postponing return to work

NA, not available; +, positive. −, negative.

## Discussion and conclusion

Currently, COVID-19 has become a pandemic worldwide. As of 4 June 2020, the cumulative number of confirmed cases has reached 6 635 295 worldwide, which has far exceeded the number of SARS and MERS infections [8]. The main symptoms of COVID-19 are fever, cough and dyspnoea. Some patients show non-influenza-like symptoms such as muscle ache, fatigue, conjunctivitis and gastrointestinal diseases [9, 10], and even more, are asymptomatic [11, 12]. The diversity of initial symptoms and asymptomatic status of COVID-19 make it more difficult to prevent and control outbreaks, especially recessive SARS-CoV-2 infection. Much less is known about the timing and pattern of viral shedding in asymptomatic infections, so asymptomatic infections may be a potential risk factor for COVID-19 re-outbreaks.

During the period from 14 May 2020 to 1 June 2020, Wuhan, China, nucleic acid test for SARS-CoV-2 of 9 898 800 residents were completed. The results showed that no confirmed cases were found, and 300 asymptomatic infections were detected, with a detection rate of 0.0303‰, which was an amazing achievement. In total, 1174 close contacts have been traced and managed, and their nucleic acid detection results were negative [13]. In our study, the detection rate of asymptomatic infections among staff returning to work in this hospital was about 0.8458‰. These data suggest that asymptomatic infections still exist in COVID-19 low-risk regions, but the number of infected individuals is small. Safety issues in the process of returning to work are the focus of attention. Therefore, SARS-CoV-2 screening of the population at a specific time and region may be an important means of curbing potential infections.

The 4729 returned staff, all from Zhongnan Hospital of Wuhan University, underwent comprehensive SARS-CoV-2 screening, including SARS-CoV-2 nucleic acid test, antibody detection, chest CT scan, body temperature measurement and recording of infection characteristics. The results showed that two patients were positive in the first nucleic acid test. In addition, one patient was negative in the initial nucleic acid test but positive after re-examination. In total, 120 employees (2.54%) had positive results for antibodies (IgG or IgM), and there was no correlation in the production of IgM and IgG antibodies among different ages, sexes and occupations. In the 2194 employees (46.39%) who had chest CT scans, 25 employees had viral pneumonia changes in their lungs and 31 employees found non-viral pneumonia lesions. In addition, during the initial physical examination, the body temperature of each returned staff was normal and there were no respiratory infection symptoms. These data suggest that a wide range of health screening will help identify recessive infections with SARS-CoV-2 in asymptomatic populations.

Nucleic acid test or gene sequencing is the gold standard for confirming SARS-CoV-2 infection, and comprehensive consideration of CT imaging and antibody detection helps to improve the detection rate [7, 14, 15]. Currently, there is still some controversy in the diagnosis of COVID-19 by chest CT scan. It is undeniable that chest CT image can reflect whether there is pulmonary infection and its severity. However, in view of this radioactive imaging technique, it cannot accurately determine which type of viral infection is and the uncertain harm of radiological technology to human body, so we think that CT scan can only be used as an important auxiliary diagnostic technology, it is of great value during the COVID-19 outbreak, but to some extent, we believe that CT scan is not suitable for large-scale health screening.

It has been found that asymptomatic people infected with SARS-CoV-2 also have many transmission routes. Respiratory virus (pharyngeal virus) shedding has been found in asymptomatic infections, confirming their potential ability to spread [16]. Jiang *et al*. [17] suggested that the faecal–oral route might be another way of transmission for asymptomatic infections. In addition, viral shedding in the blood is very common. Although some studies suggested that the possibility of blood transmission of SARS-CoV-2 is low, there is still a certain risk [18–21]. In our study, four employees were assessed as high risk and arranged for isolation treatment in designated hospital. They were asymptomatic but possibly at risk of transmission. Three employees showed low risk and suggested postponing return to work. These results suggest that there may be recessive SARS-CoV-2 infection in the population, which is a major risk factor in the process of returning to work. Close contacts were tracked and managed by the Chinese CDC, and we believe that early detection and management of potential COVID-19 cases, as well as identification and isolation of close contacts, are essential measures to curb the COVID-19 epidemic [22].

We found that SARS-CoV-2 RNA RT-PCR may have false negative result for SARS-CoV-2 infection under certain conditions, especially for asymptomatic infections. Among 172 people with abnormal first physical examination results, we observed that 170 cases (98.84%) were negative in the first SARS-CoV-2 nucleic acid test, but one of which was positive by RT-PCR at the time of reexamination. On the other hand, 120 (69.77%) of the population with abnormal first physical examination results were antibody positive, but only seven were classified as at risk of infection, and the remaining 113 (65.70%) were considered to have protective antibodies *in vivo*, and we speculate that they may have been infected with SARS-CoV-2. Usually, IgM antibodies are regarded as indicators of recent infection, generally occurring 3–7 days after infection, while IgG antibodies are produced later than IgM antibodies, but with good stability [23]. Notably, no studies have been conducted to correlate SARS-CoV-2 IgM antibody titres with transmission potential in asymptomatic populations.

### Limitations

First, this study did not investigate the personal history and epidemiological history of people with abnormal results of the first physical examination, and could not conduct in-depth study on the effective transmission ability of asymptomatic infections. Second, haematological tests have not been performed, and there is a lack of description of the clinical characteristics of people with abnormal first physical examination results.

In summary, we efficiently identified asymptomatic infections through extensive health screening of all returned staff from a hospital in a short period of time, combined with a variety of inspection methods (including nucleic acid test for SARS-CoV-2, antibody detection and chest CT scan), and emphasising the presence of asymptomatic infections in the general population. Besides, close contacts were tracked and managed by Chinese CDC, this will minimise the possibility that asymptomatic SARS-CoV-2 infections will transmit the virus in the process of returning to work. Currently, in countries or regions with stable control of COVID-19 epidemic, asymptomatic infections may be an important potential risk factor for re-outbreaks. Therefore, we believe that a wide range of health screening combined with various laboratory tests can help to identify asymptomatic infections. The spread of COVID-19 will be effectively curbed through strict isolation of treatment and development of close contact tracking programmes.

## Data Availability

The datasets generated during and/or analysed during the current study are available from the corresponding author on reasonable request.
